# Tuberculosis service utilisation pre-COVID-19 and peri-COVID-19 restrictions in Johannesburg, Gauteng

**DOI:** 10.4102/safp.v68i1.6296

**Published:** 2026-06-30

**Authors:** Vongani H. Maluleke, Lynn Smith

**Affiliations:** 1 Department of Environmental Health, Faculty of Health Sciences, University of Johannesburg, Johannesburg, South Africa; 2 Department of Sport and Movement, Faculty of Health Sciences, University of Johannesburg, Johannesburg, South Africa

**Keywords:** COVID-19, tuberculosis, TB services, health care access, public health service

## Abstract

**Background:**

Tuberculosis (TB) has remained a significant public health challenge for many years. Individuals with weakened immune systems because of other illnesses are particularly vulnerable to contracting TB. In March 2020, South Africa implemented a disease control strategy which ceased and disrupted daily routines, including healthcare services delivery. The study aimed to determine and describe the impact of coronavirus disease 2019 (COVID-19) lockdown restrictions on the utilisation of TB services, pre-COVID-19 and peri-COVID-19.

**Methods:**

A cross-sectional retrospective study using National Institute for Communicable Diseases (NICD) and National Department of Health (NDoH) data from Johannesburg, South Africa, was conducted to assess COVID-19 restrictions’ impact on TB service use from January 2018 to December 2021, examining trends across periods and influencing factors. The study included individuals who sought TB services, showed TB symptoms, or tested positive or negative for TB during the study period.

**Results:**

The secondary data collected included children (< 10 years old), young adolescents (10–14 years old), older adolescents (15–19 years old), young adults (20–24 years old), adults (25–49 years old) and older adults (≥ 50 years old). Tuberculosis service use was highest among adult females aged 25–29 years old (51.3%) and lowest among females ≥ 50 years old (32.6%).

**Conclusion:**

A marked decline in TB service use was observed during the peri-COVID-19 period. Results demonstrated that COVID-19 restrictions caused a substantial and sustained decline in TB service utilisation in Johannesburg, even when accounting for broader determinants of care-seeking.

**Contributions:**

This study provides valuable insight into the impact of COVID-19 restrictions on TB service utilisation and highlights the broader societal effects of a pandemic.

## Introduction

The coronavirus disease 2019 (COVID-19) pandemic affected everyone’s daily life, including the public health system and economy.^1,2^ When the World Health Organization (WHO) declared COVID-19 a pandemic, South Africa comprehensively upgraded its prevention measures, which prompted authorities to implement restrictions to lower disease transmission. These restrictions included physical distancing, self-isolation, closure of non-essential services and schools, travel and recurrent national lockdowns.^1,3^ This state of disaster response was subsequently categorised by levels, with Level 5 being the most severe and Level 1 being the least. In 2020, the transition from Level 5 to Level 1 occurred on 27 March, 01 May, 13 July, 19 August and 21 September, respectively.^4,5^ The different disaster levels affected individuals’ daily routines, including adherence to disease prevention measures and health-seeking behaviour.^6^

The provision of primary and hospital care was disrupted, thus tuberculosis (TB) management and all aspects of TB care were impacted, highlighting the need for an evaluation of TB services prior to and during COVID-19 to understand the trends and impact.^7^ Scheunemann et al.^8^ note that COVID-19 disrupted gains in the global TB response, having a serious impact on the most vulnerable populations. Routine healthcare services in South Africa, which already face a high burden of TB and human immunodeficiency virus (HIV), were negatively impacted during the pandemic.^9,10,11,12^

In the 2021 Global TB Report, the WHO reported an increase in TB cases and deaths from TB for the first time in decades.^13^ An estimated 10.6 million people were diagnosed with TB in 2021.^14^ Fewer cases of TB were detected, and fewer people were treated for TB during 2020/2021 because of disruptions caused by COVID-19.^14^

Reduced access to TB testing and treatment resulted in an increase in TB deaths, back to the level of 2017, along with an increase in TB incidence.^15^

Other impacts included reductions in the number of people provided with treatment for drug-resistant TB and TB preventive treatment, and a fall in global spending on TB diagnostic, treatment and prevention services.^14^ Literature has highlighted the potential impact of the COVID-19 pandemic and lockdown on patients and healthcare services in South Africa.^16,17^

Factors such as fear of contracting COVID-19 and associated stigma,^18^ restrictions on movement, increased family responsibilities, unreliable transport and loss of income or employment negatively impacted health-seeking behaviour and access to services.^19^ On the service side, the deprioritisation of routine health services, diversion of resources and repurposing of the health workforce led to a reordering of healthcare priorities, affecting both demand and supply.^20^ Therefore, the aim of this study was to assess the impact of COVID-19 restrictions on TB service utilisation in the City of Johannesburg, with the purpose of understanding how these disruptions affected access to routine TB care.

## Research methods and design

### Study design

A cross-sectional retrospective study was conducted, analysing quantitative and descriptive secondary data. The study design was used for population-based surveys and to assess the prevalence of diseases in clinic-based samples.^21^

### Setting

The study setting was the City of Johannesburg in Gauteng province, South Africa. This included data from all government hospitals and clinics within the City of Johannesburg Metropolitan District.

### Study population and sampling strategy

The study population included everyone who accessed healthcare services in Johannesburg. The sample was restricted to those who accessed TB services during our study period (January 2018 – December 2021) in the Johannesburg district.

### Data collection

The study used de-identified routine TB secondary data from Johannesburg facilities operating before and during the COVID-19 pandemic, including individuals who accessed TB services, showed symptoms, or tested positive or negative between January 2018 and January 2022. Data outside this time frame or from outside Johannesburg were excluded.

### Data analysis

Descriptive analysis was undertaken to categorise patients into children (< 10 years), young adolescents (10–14 years), older adolescents (15–19 years), young adults (20–24 years), adults (25–49 years) and older adults (≥ 50 years). Additional demographic information, including district within the Johannesburg Metropolitan Municipality, sex, race and year of diagnosis, was also collected. Patient diagnoses were classified as follows: abscess, anaemia, cough, COVID-19, drenching night sweats (DNS), haemoptysis, HIV, immunodeficiency, weight loss, lower respiratory tract infections, lymphoma, lymphadenopathy, malignancy, malaria, malnutrition, meningitis, noncommunicable diseases (NCDs), pregnancy, pleural effusion, pneumonia, sepsis and TB.

Information on demographics and diagnoses was summarised by age groups using proportions for categorical variables. Descriptive analysis was done to characterise the study setting, sample and provide an overall description of the data. A period-over-period chart was used to illustrate changes in TB screening data across the study months or period using timeframe analysis. This period over chart shows the relationship between the TB data across months and highlights important trends. Data was represented on a line graph to show the trend of screening tests over the study period. Descriptive statistical analysis was used to outline the characteristics of the dataset by summarising and presenting key features through tables, charts, and graphs of the sampled data, enabling evidence-based conclusions.

### Ethical considerations

The Institutional Research Ethics Committee of the University of Johannesburg granted ethical approval and guided the research process (REC-2920-2024).

## Results

The researchers identified 37 513 records in the City of Johannesburg between January 2018 and December 2021. These records constituted the sample of children, adolescents, young adults, adults and older adults who accessed TB services during the study period. Table 1 presents the demographic characteristics of the study sample. The sample represented 60.3% (*n* = 22 618) of cases during the pre-COVID-19 period and 39.7% (*n* = 14 894) during the peri-COVID-19 period, out of an estimated 1.4 million cases reported on the national TB surveillance platform for the Gauteng Province.

**TABLE 1:** Distribution of tuberculosis cases by demographic and clinical characteristics during the pre-COVID-19 (2018–2019) and peri-COVID-19 (2020–2021) periods in the City of Johannesburg.

Variables	Total (*N* = 37 513)		Pre-COVID-19 (*N* = 22 618)		Peri-COVID-19 (*N* = 14 895)	
	*n*	%	*n*	%	*n*	%
**Age groups**						
Children (< 10 years)	550	1.5	344	1.5	206	1.4
Young adolescent (11–14 years)	219	0.6	130	0.6	89	0.6
Older adolescents (15–18 years)	590	1.6	372	1.6	218	1.5
Young adults (19–24 years)	2775	7.4	1631	7.2	1144	7.7
Adults (25–49 years)	26 065	69.5	15 760	69.7	10 305	69.2
Older adults (≥ 50 years)	5754	15.3	3376	14.9	2378	16.0
Unspecified age	1560	4.2	1005	4.4	555	3.7
**Sex**						
Male	22 191	59.2	13 239	58.5	8952	60.1
Female	14 205	37.9	8697	38.5	5508	37.0
Unknown	1117	3.0	682	3.0	435	2.9
**Registration year**						
2018	12 177	32.5	12 177	53.8	0	0
2019	10 441	27.8	10 441	46.2	0	0
2020	7609	20.3	0	0	7609	51.1
2021	7286	19.4	0	0	7286	48.9
**Region**						
Johannesburg A	3578	9.5	2075	9.2	1503	10.1
Johannesburg B	4362	11.6	2644	11.7	1718	11.5
Johannesburg C	2089	5.6	1280	5.7	809	5.4
Johannesburg D	13 120	35.0	7979	35.3	5141	34.5
Johannesburg E	3657	9.7	2257	10.0	1400	9.4
Johannesburg F	6354	16.9	3915	17.3	2439	16.4
Johannesburg G	4353	11.6	2468	10.9	1885	12.7
**Clinical diagnosis**						
Abscess	116	0.3	57	0.3	59	0.4
Anaemia	3	0.0	3	0.0	0	0.0
Cough	105	0.3	72	0.3	33	0.2
COVID-19	11	0.0	0	0.0	11	0.1
Drenching night sweats (DNS)	639	1.7	237	1.0	402	2.7
Haemoptysis	9	0.0	4	0.0	5	0.0
Human immunodeficiency virus (HIV)	57	0.2	38	0.2	19	0.1
Immunodeficiency	12	0.0	7	0.0	5	0.0
Renal artery stenosis (RVD)	85	0.2	77	0.3	8	0.1
Lower respiratory tract infections (LRTI)	108	0.3	54	0.2	54	0.4
Lymphoma	27	0.1	27	0.1	0	0.0
Lymphadenopathy	10	0.0	1	0.0	9	0.1
Malignancy	1	0.0	1	0.0	0	0.0
Malaria	1	0.0	1	0.0	0	0.0
Malnutrition	1	0.0	0	0.0	1	0.0
Meningitis	82	0.2	55	0.2	27	0.2
Noncommunicable disease (NCDs)	10 022	26.7	6717	29.7	3305	22.2
Pregnancy	3	0.0	0	0.0	3	0.0
Pleural effusion	61	0.2	44	0.2	17	0.1
Pneumonia	71	0.2	58	0.3	13	0.1
Sepsis	18	0.0	11	0.0	7	0.0
TB	3644	9.7	3048	13.5	596	4.0
Non specified	22 427	59.8	12 106	53.5	10 321	69.3

COVID-19, coronavirus disease 2019; TB, tuberculosis.

### Age distribution

The age distribution of participants was summarised using categorical age groups. The majority of individuals (69.5%) fell within the 25–49-year age group, representing the largest proportion of TB service users in the dataset. This was followed by participants aged 50 years and above (15.3%), young adults aged 19–24 years (7.4%), and those with unspecified age information (4.2%). Smaller proportions were observed among older adolescents aged 15–18 years (1.6%), children under 10 years (1.5%) and young adolescents aged 11–14 years (0.6%).

Because age was captured in categories rather than as continuous values, measures of central tendency, such as mean and standard deviation, are not appropriate. Instead, the distribution is best characterised by proportions across the predefined age groups. Visual inspection of the age-group frequency distribution indicates that the data are left-skewed, with the highest concentration of cases among adults aged 25–49 years.

### Sex and race distribution

Males represented 59.2% of the sample, while females accounted for 37.9%; 3.0% had unspecified sex information. Racial identifiers were largely unavailable, with 99.8% of participants recorded as ‘unknown’. This limits meaningful interpretation of race-related patterns.

### Tuberculosis service use by year

Across the four study years, the highest number of TB cases was recorded in 2018 (32.5%), followed by 2019 (27.8%). Service utilisation declined sharply during the COVID-19 pandemic, with 20.3% of cases recorded in 2020 and 19.4% in 2021. This decline corresponds with the onset of national COVID-19 restrictions and disruptions to routine healthcare services.

### Geographical distribution

Tuberculosis service utilisation varied across the seven administrative regions of the City of Johannesburg. Region D reported the highest proportion of cases (35.0%), followed by Region F (16.9%) and Regions B and G (11.6% each), while Region C reported the fewest cases (5.6%). While these differences may reflect variation in population density, facility distribution and service accessibility, the relative decline in TB patient attendance following the onset of COVID-19 was consistent across all regions, ranging between 38% and 42%. This uniformity suggests that the disruption to TB services was widespread and systemic, affecting service utilisation across the metropolitan area regardless of regional differences.

### Clinical presentation

Participants presented with a diverse range of diagnoses prompting TB screening. The largest proportion (59.8%) fell under ‘non-specified’ diagnoses, followed by individuals with non-communicable diseases (26.7%). Those already diagnosed with TB accounted for 9.7% of cases, while smaller proportions (< 1%) were associated with conditions such as DNS, pneumonia, pleural effusion and meningitis.

When comparing the pre-COVID-19 and peri-COVID-19 periods, several conditions demonstrated a marked decline, including renal artery stenosis, lymphoma, pneumonia and TB. This reduction may reflect decreased healthcare utilisation, limited access to diagnostic services such as imaging and specialist consultations, and shorter or prioritised clinical encounters during the pandemic. In contrast, an increase in lymphadenopathy was observed, which may be attributable to its role as a clinically detectable sign requiring minimal diagnostic resources, as well as heightened vigilance for infectious or inflammatory conditions during the COVID-19 period.

As only individuals who tested positive for TB were included in this study, these trends are interpreted with caution, as the dataset does not capture patients who screened negative or were not tested.

**FIGURE 1:**
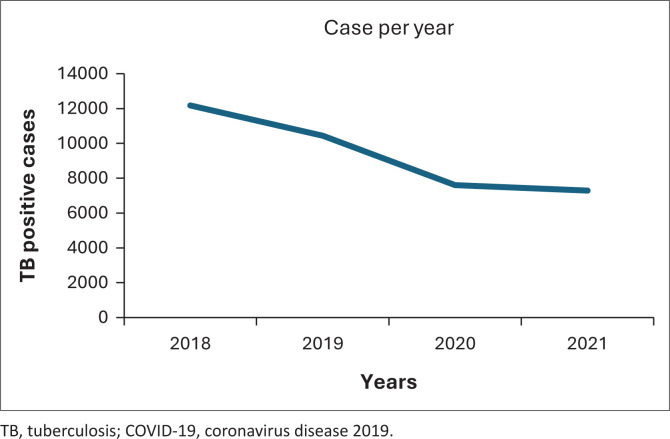
Annual tuberculosis-positive case counts showing a marked reduction between the pre-COVID-19 and peri-COVID-19 periods.

Figure 1 shows the annual distribution of TB cases recorded in the dataset, with an overall decline of 4891 cases across the study period. Notably, a decrease of approximately 1900 cases is observed between 2018 and 2019, prior to the onset of the COVID-19 pandemic. The reasons for this pre-pandemic decline are not fully clear and may reflect underlying health system challenges, changes in case detection practices, or natural variation in TB reporting trends. A further decline between 2019 and 2020 coincides with the onset of the COVID-19 pandemic and is more plausibly linked to disruptions in healthcare access, including movement restrictions, fear of infection, and the reallocation of healthcare resources.

Data beyond the study period were not available to assess the recovery of TB service utilisation following the COVID-19 pandemic, which represents an important area for future research.

## Discussion

This study examined the impact of COVID-19 restrictions on TB service utilisation in the City of Johannesburg. The findings reveal a substantial decline in the number of individuals accessing TB services during the peri-COVID-19 period compared with the pre-pandemic years. This decline occurred despite the high burden of TB in South Africa, suggesting that COVID-19 response measures significantly disrupted routine healthcare access. However, a decline in TB service utilisation was also observed in the 2 years preceding the COVID-19 pandemic, indicating that the downward trend was not solely attributable to COVID-19-related disruptions. The reasons for this pre-pandemic decline are not fully clear and may reflect underlying health system challenges, changes in case detection and reporting practices, or natural variation in service utilisation.^22^ While structural factors such as the burden of HIV, non-communicable diseases, and socio-economic barriers are known to influence TB care,^22^ these were not directly examined in the present study and should therefore be interpreted with caution.

Health-seeking behaviour is influenced by a complex interplay of individual, social, and systemic factors.^23^ In South Africa, these determinants include gender norms, socioeconomic status, health literacy and the organisation of health services.^22^ Traditionally, women are more likely to seek healthcare compared to men.^24^ However, in this study, males accounted for most TB service users (59.2%), suggesting that TB symptom presentation, occupational exposure, mobility patterns or social roles may have shaped service utilisation differently for TB than for general health services.

Variations across age groups also align with previous evidence indicating that adults bear the greatest TB burden, but there is insufficient morbidity data on HIV and/or TB among older adults.^25^ Consistent with this, adults aged 25–49 years constituted nearly 70% of the sample, while children and adolescents were underrepresented.

The study also observed a high proportion of individuals with non-specified diagnoses and NCDs accessing TB services. Tuberculosis–noncommunicable disease multimorbidity is increasingly recognised as an important public health issue, as chronic diseases such as diabetes and cardiovascular conditions can increase susceptibility to TB infection and worsen treatment outcomes.^26^ The interaction between HIV, TB, and COVID-19 further complicates disease presentation and health-seeking behaviour, as co-infected individuals may delay care because of fear of exposure to COVID-19, stigma or restricted mobility.^27^

The implementation of COVID-19 restrictions coincided with a pronounced decline in TB service utilisation. The strictest alert level (Level 5) was associated with the steepest decrease in TB testing and diagnosis, consistent with national and global reports of disruptions in essential health services.^11^ Reduced mobility, fear of contracting COVID-19, diversion of healthcare resources, and closure or scaling down of routine services all contributed to this decline.^28^

The gradual recovery observed as restrictions eased suggests that the decline was not because of a true reduction in TB incidence but rather because of barriers in accessing care. Nevertheless, the number of TB cases diagnosed during Level 1 remained significantly lower than pre-pandemic levels, indicating persistent challenges in health-seeking behaviour and service availability.

These findings reinforce earlier studies demonstrating that the COVID-19 pandemic reversed gains in TB detection, treatment initiation and continuity of care.^8^ The impact appears particularly pronounced in high-burden settings such as South Africa, where health systems were already strained by dual epidemics of HIV and TB. To mitigate similar disruptions in the future, several strategies should be considered. The observed system-wide decline in service utilisation suggests the need for resilient, integrated service delivery models that can sustain essential TB services alongside emergency responses.

### Implications for tuberculosis control and health systems resilience

The disruption of TB services observed in this study, including the consistent decline in utilisation across all regions, underscores the need for resilient health systems capable of maintaining essential services during widespread public health emergencies. The uniformity of this decline suggests that future interventions should be implemented at a system-wide level rather than targeting specific geographic areas.

Integrated service delivery models, such as combined TB, HIV and COVID-19 screening, together with multi-month dispensing and community-based medication delivery, may help to sustain continuity of care during similar disruptions. In addition, the observed reduction in facility-based attendance highlights the potential role of telemedicine platforms to support patient follow-up, symptom monitoring and adherence counselling without requiring in-person visits.

The large proportion of ‘non-specified’ diagnoses in the dataset further highlights gaps in routine health information systems. While this limits diagnosis-specific interpretation, it does not substantially affect the observed patterns of service utilisation. However, it does underscore the need for improved clinical documentation and diagnostic coding practices to enhance the value of routine data for service planning, monitoring, and future research.

### Strengths

This study provides valuable insight into the impact of COVID-19 restrictions on TB service utilisation and highlights the broader societal effects of a pandemic. By analysing service utilisation across different lockdown alert levels, the study offers a nuanced understanding of how varying restrictions influenced TB care. It emphasises the importance of out-of-care, community-based interventions that can sustain TB service utilisation during public health emergencies and beyond, particularly for TB patients and those co-infected with other conditions. The descriptive analysis provides insight into the study sample and service utilisation patterns, while the findings underscore the importance of maintaining continuity of care for TB and other chronic conditions during pandemics. In line with existing literature, the results also highlight the influence of social determinants on access to and provision of TB services.

### Limitations

As with other studies relying on secondary data, this research faced limitations related to data availability and quality. Missing or incomplete data constrained the study’s ability to fully explore TB trends and service utilisation patterns during the pandemic. Inconsistencies in identity numbers and constant variables across the pre-COVID-19 and peri-COVID-19 periods limited the capacity to establish associations between variables. Despite these constraints, the study provides meaningful insights into the effects of pandemic restrictions on routine TB services.

## Conclusion

Health-seeking behaviour is shaped by complex individual, social and systemic factors. Our findings demonstrate that COVID-19 restrictions caused a substantial and sustained decline in TB service utilisation in Johannesburg, even when accounting for broader determinants of care-seeking. These disruptions reveal vulnerabilities in the health system and underscore the urgent need for innovative, community-based strategies that maintain continuity of care for TB and at-risk populations during public health emergencies. Integrated, multidisciplinary approaches addressing TB, HIV, non-communicable diseases and COVID-19 are essential to strengthen service delivery and sustain health-seeking behaviour.

Moreover, investing in centralised, comprehensive health data systems is critical. Timely access to robust data will support evidence-based policymaking, guide targeted interventions and drive systemic improvements in healthcare delivery. Strengthening adaptable, community-focused and patient-centred health services is vital – not only to manage future public health crises but also to ensure ongoing TB control and reduce preventable morbidity and mortality.
